# Risk of ischemic and hemorrhagic complications associated with continuation versus interruption of antithrombotic therapy for cervical transforaminal injections: A retrospective study

**DOI:** 10.1016/j.inpm.2026.100820

**Published:** 2026-09-08

**Authors:** Audrey V. Adler, Daniel C. Persson, Christopher G. Leonardi, Cody S. Crandall, Jessica C. Garcia, William Tang, Akbar S. Nabi, Amanda N. Cooper, Katharine A. Smolinski, Zachary L. McCormick, Aaron M. Conger

**Affiliations:** Department of Physical Medicine and Rehabilitation, University of Utah, Salt Lake City, UT, USA

**Keywords:** Cervical transforaminal epidural steroid injection, Cervical selective nerve root block, Epidural hematoma, Antithrombotic therapy

## Abstract

**Background:**

Cervical transforaminal epidural steroid injections (CTFESI) and cervical selective nerve root blocks (CSNRB) are commonly performed for the diagnosis and treatment of cervical radicular pain. Periprocedural management of antithrombotic therapy remains challenging, requiring a balance between the potential risk of hemorrhagic complications, particularly epidural hematoma, and the risk of thromboembolic complications associated with medication interruption. Although recent evidence suggests a low risk of hemorrhagic complications when antithrombotic therapy is continued during spinal procedures, data specific to cervical transforaminal injections remain limited.

**Objective:**

To assess the incidence of ischemic and hemorrhagic complications at 0—30 days (primary outcome) and 31—90 days (secondary outcome) post-procedure among patients undergoing CTFESI and CSNRB who continued versus interrupted antithrombotic therapy prior to the procedure.

**Methods:**

Retrospective chart review of consecutive patients who underwent fluoroscopically-guided CTFESI or CSNRB at a single tertiary academic institution.

**Results:**

A total of 105 procedures from 95 patients met inclusion criteria. Antithrombotic therapy was continued in 25 procedures (23.8%) and interrupted in 80 procedures (76.2%). No hemorrhagic complications occurred in either group from 0 to 30 days (continued: 0/25, 0.0%, 95% CI 0.0—13.3; interrupted: 0/80, 0.0%, 95% CI 0.0—4.6; *p* = 1.000). Ischemic complications occurred from 0 to 30 days in 1 procedure in the continued group (1/25, 4.0%, 95% CI 0.7-19.5) and 2 procedures in the interrupted group (2/80, 2.5%, 95% CI 0.7-8.8; *p* = 0.562). No additional hemorrhagic or ischemic complications occurred between 31 and 90 days in either group.

**Conclusion:**

No hemorrhagic complications were observed following cervical transforaminal injections when antithrombotic therapy was continued or interrupted, though 3 total ischemic events occurred. Due to the small sample size and low frequency of observed events, this study provides primarily descriptive data on adverse events rather than offering definitive evidence for procedure safety when continuing therapy or comparative ischemic risk between groups. Larger studies are needed to better characterize the risks associated with continuation versus interruption of antithrombotic therapy.

## 1 Introduction

Cervical transforaminal epidural steroid injections (CTFESI) are a commonly performed, minimally invasive treatment option for patients with cervical radicular pain. They provide targeted delivery of a corticosteroid to the affected nerve root to reduce inflammation and alleviate symptoms [[1], [2], [3], [4]]. Cervical selective nerve root block (CSNRB) procedures are similar in technique but are typically performed for diagnostic rather than therapeutic purposes, utilizing smaller volumes of injectate with the goal of limiting spread into the epidural space and targeting specific nerve roots [5,6]. Cervical transforaminal injections carry a rare but recognized risk of bleeding complications, including symptomatic epidural hematoma [7,8]. These events are hypothesized to result from injury to the posterior internal vertebral venous plexus, a vascular network that is susceptible to trauma during needle advancement [[9], [10], [11], [12]]. Complications include neurological deficits that range in severity depending on initial symptoms and time to surgical decompression [13].

Concern for the risk of major bleeding complications including epidural hematoma is reflected in earlier guidelines, such as those published by the American Society of Regional Anesthesia and Pain Medicine (ASRA) in 2015 and updated in 2018. These guidelines categorized CTFESI as intermediate-risk procedures and recommended withholding most anticoagulant and antiplatelet medications before the procedure [14,15].

However, emerging evidence has begun to challenge the assumption that interruption of antithrombotic therapy truly represents the conservative approach. Anticoagulants and antiplatelet agents, collectively referred to as antithrombotic therapy, are commonly prescribed for atrial fibrillation, venous thromboembolism, mechanical heart valves, coronary artery disease, peripheral arterial disease, and prior ischemic stroke, all of which are independently associated with elevated rates of future thromboembolic complications [[16], [17], [18], [19]]. Studies from interventional spine, interventional pain, and other procedural disciplines have found an increased risk of thromboembolic complications associated with antithrombotic therapy interruption [[20], [21], [22], [23], [24], [25], [26]].

Recent cohort studies and systematic reviews have found an extremely low risk of clinically significant bleeding complications among patients continuing antithrombotic medications during other common spine injections. The most common procedures included in these prior studies were lumbar injections (both transforaminal epidural steroid injections and facet injections), medial branch blocks and radiofrequency neurotomies at cervical and lumbar levels, and sacroiliac joint injections [9,20,21,[27], [28], [29], [30], [31], [32]].

Despite these reassuring data, important gaps remain, particularly with respect to cervical spine procedures [33]. Compared with lumbar interventions, there is relatively limited data evaluating the safety of continuing antithrombotic therapy during CTFESI and CSNRB. While sparse, emerging evidence supports the safety of continuing antithrombotic therapy during CTFESI [34,35]. Additionally, several larger-scale studies evaluating the safety of cervical transforaminal injections in general (where antithrombotic therapy management is not specified) have reported zero cases of clinically significant bleeding events or spinal hematomas following CTFESI and CSNRB [[36], [37], [38]].

The purpose of this study was to assess the incidence of hemorrhagic and ischemic complications among patients who either continued or interrupted antithrombotic therapy before undergoing CTFESI and CSNRB.

## 2 Methods

### 2.1 Participants and data collection

This retrospective chart review study was approved by the University of Utah Institutional Review Board (IRB 00072431) and evaluated patients who underwent CTFESI or CSNRB at a single, tertiary academic institution between January 2013 and December 2025. The EMR was queried for patients identified using CPT code 64479 who were concomitantly consuming any of the following medications within 3 months prior to the procedure date: aspirin, Excedrin, edoxaban, apixaban, rivaroxaban, dabigatran, enoxaparin, dalteparin, heparin, pentosan polysulfate sodium, clopidogrel, cilostazol, dipyridamole, prasugrel, ticagrelor, ticlopidine, aspirin/dipyridamole, or warfarin.

Consecutive medical records were retrieved and screened for eligibility. Patients were included if they met the following criteria: (1) underwent CTFESI or CSNRB, (2) had documentation of active antithrombotic therapy at the time of the scheduled procedure, including documentation sufficient to determine whether this therapy was interrupted or continued, and (3) had at least 90 days of available follow-up in the electronic health record after their procedure. Each CTFESI or CSNRB procedure was treated as a distinct encounter. Antithrombotic therapy included anticoagulant and antiplatelet medications. The decision to hold versus continue antithrombotic therapy was based on clinical judgment of the interventionalist.

Antithrombotic therapy management was categorized as interrupted or continued based on guidelines from the 2018 American Society of Regional Anesthesia and Pain Medicine (ASRA) [15]. To determine whether antithrombotic therapy met criteria for continued versus interrupted, pre-procedural documentation associated with the procedure encounter was reviewed. This included either a standardized pre-procedure nursing checklist that records patients’ responses to whether they are actively taking antithrombotic medication and the date of their last dose or communication between provider/staff and patient addressing medication status and number of days held documented in messaging, telephone encounters, or office notes. Patients were excluded if they denied active antithrombotic therapy or if documentation was absent of insufficient to determine whether therapy was held or continued.

For warfarin, the ASRA guidelines recommend warfarin should be discontinued for 5 days and the international normalized ratio (INR) normalized (≤1.2) before intermediate- and high-risk pain procedures. Consistent with these recommendations, warfarin was classified as interrupted if the preprocedural INR was ≤1.2 or, when no recent INR was available, if the medication had been discontinued for ≥5 days before the procedure. Warfarin was classified as continued if the day-of-procedure INR was >1.2 or, in the absence of INR data, if it had been held for <5 days.

Records reviewed to assess clinically significant adverse events and determine whether therapy was interrupted or continued included CTFESI or CSNRB procedure notes, preprocedural nursing notes, outpatient clinic notes, telephone encounters, electronic health record messages, telemedicine visits, hospital admissions, and emergency department encounters. Data collected included patient age, gender, body mass index (BMI), race, ethnicity, procedure laterality and cervical level(s) targeted, antithrombotic medication(s) and indication(s) for therapy (up to 3), and whether therapy was interrupted or continued.

Cervical transforaminal epidural steroid injections and CSNRB procedures were performed under fluoroscopic guidance according to the International Pain and Spine Intervention Society guidelines by providers from the Departments of Physical Medicine & Rehabilitation, Anesthesia Pain Medicine, or Neurosurgery [39,40].

### 2.2 Outcomes

The primary outcome was the occurrence of a hemorrhagic or ischemic complication from 0 to 30 days post-procedure, while the secondary outcome was the occurrence of such an event from 31 to 90 days post-procedure. Hemorrhagic complications included clinically significant bleeding events such as epidural or paraspinal hematoma, or other bleeding complications requiring medical intervention. Ischemic complications included thromboembolic events such as stroke, transient ischemic attack, myocardial infarction, stent thrombosis, or arterial or venous thromboembolism. A 30-day follow-up window was selected for the primary outcome based on prior literature demonstrating an increased risk of thromboembolic events within 30—40 days following interruption or modification of antithrombotic therapy. This timeframe also facilitates attribution of adverse events to periprocedural antithrombotic medication management strategies as opposed to events that may be more likely to occur based on natural history independent of a spinal injection and associated change (or lack of change) in antithrombotic medication management [[22], [23], [24], [25],^41^]. Additional secondary outcomes included between-group comparisons of the number, level, and laterality of cervical sites targeted, antithrombotic medication types, and indications for antithrombotic therapy.

### 2.3 Statistical analysis

Patient demographic, clinical, and procedural variables were summarized using descriptive statistics, with calculation of means/standard deviations for continuous variables and frequencies/percentages for categorical variables. The primary and secondary outcomes were evaluated as incidences of ischemic and hemorrhagic complications within the time frames of 0–30 days and 31–90 days, respectively, with calculation of 95% confidence intervals (CIs). Between-group comparisons were based on t-tests for continuous data and chi-square tests (or Fisher's exact test in cases where expected frequencies were ≤5) for categorical data. All analyses were conducted using Stata version 18.0 (StataCorp, LLC, College Station, TX, United States) with a two-sided alpha level of 0.05.

## 3 Results

Consecutive medical records from 348 patients who underwent CTFESI or CSNRB from January 2013 to December 2025 were retrieved and screened for eligibility. Of these, 105 procedures from 95 patients met study inclusion criteria. Antithrombotic therapy was continued for 25 procedures (23.8%) from 24 patients and was interrupted for 80 procedures (76.2%) from 71 patients. Baseline demographic and procedural characteristics were similar between groups (Table 1). The mean age was 64.0 ± 12.4 years in the continued group and 64.7 ± 11.9 years in the interrupted group (*p* = 0.811). The mean body mass index (BMI) was similar between groups (29.9 ± 6.5 vs. 30.5 ± 6.1 kg/m^2^, *p* = 0.671). Patients in both groups were predominantly male (58.3% vs. 59.2%, *p* = 0.944) and identified as “White” (95.8% vs. 87.5%, *p* = 0.707). In both the continued and interrupted groups, unilateral injections and single level injections comprised the majority of procedures (unilateral 88.0% vs. 78.8%, *p* = 0.391; single-level 68.0% vs. 68.8%, *p* = 0.944).

**Table 1:** Patient demographic, clinical, and procedural variables (*N* = 95 patients; 105 procedures).

Variable	Continued (*n* = 25) *N* (%)	Interrupted (*n* = 80) *N* (%)	*p*
Gender (*n* = 95)			
Female	10 (41.7)	29 (40.9)	0.944
Male	14 (58.3)	42 (59.2)	
Ethnicity (*n* = 95)			
Hispanic	0 (0.0)	4 (5.9)	0.570
Not Hispanic	24 (100.0)	64 (94.1)	
*Unknown*	*0 (0.0)*	*2*	
Race (*n* = 95)			
Black	1 (4.2)	4 (6.3)	0.707
White	23 (95.8)	56 (87.5)	
Other	0 (0.0)	4 (6.3)	
*Unknown*	*0*	*7*	
Laterality (*n* = 105)			
Unilateral	22 (88.0)	63 (78.8)	0.391
Bilateral	3 (12.0)	17 (21.3)	
Number of levels treated (*n* = 105)			
1	17 (68.0)	55 (68.8)	0.944^a^
2	8 (32.0)	25 (31.3)	

Age (years; *n* = 105) [mean ± SD]	64.0 ± 12.4	64.7 ± 11.9	0.811^b^
BMI (kg/m^2^; *n* = 104) [mean ± SD]	29.9 ± 6.5	30.5 ± 6.1	0.671^b^

Note: Missing values are excluded from calculations. *P*-values are from Fisher's exact test, unless specified otherwise.

Abbreviations: BMI = body mass index; SD = standard deviation.

a From *x*^*2*^ test.

b From two-sample *t*-test with unequal variance.

Primary and secondary outcomes are summarized in Table 2. No hemorrhagic complications were observed in either group from 0 to 90 days (continued 0/25, 0.0%, 95% CI 0.0—13.3 versus interrupted 0/80, 0.0%, 95% CI 0.0—4.6). For the primary outcome, from 0 to 30 days, ischemic complications were observed following 1/25 procedures (4.0%, 95% CI 0.7—19.5) in the continued group and 2/80 procedures (2.5%, 95% CI 0.7—8.8) in the interrupted group. Although the observed values did not differ significantly for either category (*p = *1.000 for hemorrhagic events*; p* = 0.562 for ischemic events), the study was underpowered to detect whether a true difference exists between groups.

**Table 2:** Primary and secondary study outcomes.

Time period	Complication type	Continued (*n* = 25)		Interrupted (*n* = 80)		*p*
		*N* (%)	95% CI	*N* (%)	95% CI	
**0–30 days**	Ischemic	1 (4.0)	0.7, 19.5	2 (2.5)	0.7, 8.7	0.562
	Hemorrhagic	0 (0.0)	0.0, 13.3	0 (0.0)	0.0, 4.6	—
**31–90 days**	Ischemic	0 (0.0)	0.0, 13.3	0 (0.0)	0.0, 4.6	—
	Hemorrhagic	0 (0.0)	0.0, 13.3	0 (0.0)	0.0, 4.6	—

Note: *P*-value from Fisher's exact test.

Abbreviation: CI = confidence interval.

Regarding ischemic events, in the continued group, the sole event was unstable angina requiring coronary stent placement 13 days post-CTFESI in a patient with a history of atrial fibrillation and non-obstructive coronary artery disease whose rivaroxaban was continued. In the interrupted group, the first ischemic complication was a coronary artery bypass graft (CABG) occlusion requiring stent placement at 21 days post-CTFESI after aspirin monotherapy was interrupted. The second adverse event in this group was a pulmonary embolism (PE) in a patient with a history of a CABG whose aspirin monotherapy was held. The PE was identified on the day of the procedure because the interventionalist recommended that the patient seek additional work-up for hypoxia noted during the procedure.

The distribution of cervical levels targeted was similar between the continued and interrupted antithrombotic therapy groups (*p* = 0.458), as seen in Table 3. The distribution of antithrombotic medications was generally similar between groups with one notable exception (Table 4). Direct oral anticoagulants (DOACs) were interrupted significantly more often than they were continued (38.8% vs. 8.0%, *p* = 0.004). The indications for antithrombotic therapy were largely similar between groups (Table 5).

**Table 3:** Cervical level(s) targeted.

Cervical level	Continued (*n* = 33 levels) *N* (%)	Interrupted (*n* = 105 levels) *N* (%)	*p*
C2–C3	0 (0.0)	1 (1.0)	0.478
C3–C4	5 (15.2)	6 (5.7)	
C4–C5	8 (24.2)	22 (21.0)	
C5–C6	9 (27.3)	41 (39.0)	
C6–C7	10 (30.3)	29 (27.6)	
C7–T1	1 (3.0)	6 (5.7)	

Note: *P*-value from Fisher's exact test.

**Table 4:** Antithrombotic medications continued or held.

Medications	Continued (*n* = 25) *N* (%)	Interrupted (*n* = 80) *N* (%)	Total *N*	*p*
ASA 81 mg once daily	12 (48.0)	30 (37.5)	42	0.350^a^
ASA 81 mg twice daily	2 (8.0)	2 (2.5)	4	0.239
ASA 325 mg once daily	0 (0.0)	4 (5.0)	4	0.570
DOAC	2 (8.0)	31 (38.8)	33	**0.004**^a^
Other Antiplatelet	4 (16.0)	7 (8.8)	11	0.288
Enoxaparin (therapeutic)	1 (4.0)	7 (8.8)	8	0.677
Enoxaparin (prophylactic)	0 (0.0)	1 (1.3)	1	1.000
Warfarin	8 (32.0)	14 (17.5)	22	0.120^a^
ASA + NSAID	0 (0.0)	4 (5.5)	4	0.570
ASA + NSAID + DOAC	0 (0.0)	1 (1.3)	1	1.000
ASA + Other Antiplatelet	3 (12.0)	4 (5.0)	7	0.353
ASA + DOAC	0 (0.0)	2 (2.5)	2	1.000
ASA + Warfarin + Enoxaparin (therapeutic)	0 (0.0)	1 (1.3)	1	1.000
Warfarin + Enoxaparin (therapeutic)	1 (4.0)	7 (8.8)	8	0.677
DOAC + Other Antiplatelet	0 (0.0)	2 (2.5)	2	1.000

**Total [*N*]**	33	117	—	—

Note: DOACs included rivaroxaban and apixaban. Other antiplatelets included clopidogrel, ticagrelor, and prasugrel. NSAIDs included celecoxib and ibuprofen. *P*-values are from Fisher's exact test, unless specified otherwise. Bold font denotes statistical significance at *p* < 0.05.

Abbreviations: ASA = aspirin; DOAC = direct oral anticoagulant; NSAID = non-steroidal anti-inflammatory drug.

a From *x*^*2*^ test.

**Table 5:** Indication for antithrombotic therapy.

Indication	Continued (*n* = 25) *N* (%)	Interrupted (*n* = 80) *N* (%)	Total *N*	*p*
Atrial fibrillation/arrythmia	5 (20.0)	20 (25.0)	25	0.608^a^
Myocardial infarction	2 (8.0)	2 (2.5)	4	0.239
Coronary artery disease (CAD)	7 (28.0)	12 (15.0)	19	0.149
Valve replacement	0 (0.0)	2 (2.5)	2	1.000
Coronary artery bypass graft	0 (0.0)	4 (5.0)	4	0.570
Stent	4 (16.0)	7 (8.8)	11	0.288
Cerebral vascular accident (CVA)	3 (12.0)	9 (11.3)	12	1.000
Transient ischemic accident (TIA)	0 (0.0)	3 (3.8)	3	1.000
Pulmonary embolism (PE)	4 (16.0)	11 (13.8)	15	0.751
Deep vein thrombosis (DVT)	3 (12.0)	15 (18.8)	18	0.553
Prophylaxis against thrombotic events	3 (12.0)	3 (3.8)	6	0.145
Hypercoagulable state	2 (8.0)	4 (5.0)	6	0.627
Not specified	1 (4.0)	5 (6.3)	6	1.000
Other	1 (4.0)	7 (8.8)	8	0.677

**Total [*N*]**	35	104	—	—

Note: *P*-values are from Fisher's exact test, unless specified otherwise.

a From *x*^*2*^ test.

## 4 Discussion

This study describes the observed incidence of hemorrhagic and ischemic complications following interruption versus continuation of antithrombotic therapy among patients undergoing CTFESI and CSNRB procedures. No hemorrhagic complications occurred among the 25 procedures where antithrombotic therapy was continued, nor in the 80 procedures where therapy was interrupted. While these observations align with prior findings of a low incidence of significant hemorrhagic complications following cervical transforaminal injections, the confidence interval for the continued-group was wide (95% CI 0.0—13.3) and limits conclusions. Specifically, the absence of observed significant hemorrhagic events in the continued-group does not provide definitive evidence that the continuation of antithrombotic therapy has a low risk of hemorrhagic complications or is safe in all patients. Instead, these findings add descriptive data to the limited existing literature.

Regarding prior studies demonstrating a low observed rate of hemorrhagic complications, no clinically significant bleeding events were reported in two retrospective cohorts of patients undergoing CTFESI while continuing antithrombotic medications, including 14 patients in one study and 277 patients receiving anticoagulants and/or aspirin in another, which also included 763 patients continuing nonsteroidal anti-inflammatory drugs (NSAIDs) [34,35]. Additional retrospective studies that have not specified periprocedural antithrombotic management also support the safety of fluoroscopically-guided CTFESI. No major hemorrhagic complications were reported among 6241 procedures by Beckworth et al. or in a separate cohort of 1752 procedures by Lee et al. [37,38] A retrospective cohort study of 72,125 CTFESI procedures identified from a large national administrative claims database was unable to quantify the incidence of epidural hematomas because fewer than 11 events occurred, providing additional evidence that this complication is exceedingly rare [42]. A prospective study that included both CT-fluoroscopic guidance and fluoroscopically-guided procedures reported no hemorrhagic complications among 1408 cervicothoracic transforaminal epidural steroid injections, although the distribution of cervical versus thoracic injections was not specified [36]. Another prospective study of 799 CSNRB procedures reported no serious hemorrhagic complications [43].

However, there is evidence that the risk of serious hemorrhagic complications associated with CTFESI is non-zero. A recent retrospective study of patients identified from a large, national multi-insurance administrative claims database reported 18 cases of epidural hematoma among 220,597 patients who received a cervicothoracic transforaminal epidural injection, corresponding to an incidence of 0.008% [7]. However, the study could not differentiate cervical from thoracic procedures and did not report whether fluoroscopic guidance was utilized or whether injections were performed using appropriate procedural technique [40]. Additionally, Cho et al. describe a case report of a patient taking aspirin and clopidogrel who developed a C4-C6 epidural hematoma following a fluoroscopically-guided C4-C5 CTFESI. The epidural hematoma was managed non-operatively due to improvement with conservative care [8]. A separate case report by Lee et al. describes a patient who presented 4 days after a reported right C7-T1 TFESI with severe upper thoracic pain and progressive loss of sensation in the lower extremities. MRI revealed a T1-T5 epidural hematoma that required surgical decompression. The patient reportedly had not been taking anticoagulants, aspirin, or NSAIDs. Within 6 months, the patient regained full strength and sensation [44]. However, the ability to draw meaningful conclusions from the information presented in this case report has been questioned given that no procedural images were provided and because it does not appear that the authors were proceduralists [45].

In our study, there were two ischemic complications from 0 to 30 days post-procedure among the 80 cases in which antithrombotic therapy was interrupted and one complication among the 25 procedures where therapy was continued. The incidence of observed complications was 2.5% (95% CI 0.7—8.8) in the interrupted group and 4.0% (95% CI 0.7—19.5) in the continued group. Although this difference was not statistically significant (*p* = 0.562), our study was not powered to determine whether a true difference in the rate of ischemic complication exists between groups. The uncertainty around these estimates is evidenced by the wide confidence intervals. These findings should be viewed as descriptive observations rather than provide definitive evidence that the groups have equal complication rates. There were no ischemic complications from day 31—90.

Our findings suggest that patients on antithrombotic therapy undergoing procedures remain at risk for thromboembolic complications. For example, in our study two patients had thromboembolic events that are not typically prevented by the therapy that was either interrupted or continued. One patient with a history of a CABG developed a pulmonary embolism after aspirin was interrupted. This was diagnosed the same day of the injection per recommendation that the patient seek further evaluation for new hypoxia noted throughout the procedure. Aspirin monotherapy is not routinely used for primary treatment nor prophylaxis for thrombotic events, although its role in venous thromboembolism prevention, especially in the context of major orthopedic surgery, is currently debated [[46], [47], [48]]. Similarly, a patient who continued rivaroxaban developed unstable angina requiring stent placement 13 days post-procedure. Typically, coronary artery disease is managed with antiplatelet agents rather than anticoagulants like DOACs [49,50]. Conversely, the third event was more closely associated with the type of antithrombotic therapy that was interrupted. Specifically, a patient with a prior (>1 year) history of CABG developed an occlusion of the graft requiring stent placement after aspirin monotherapy was interrupted. This occurred 21 days post-CTFESI. Together, these events demonstrate that patients on antithrombotic therapy remain vulnerable to thromboembolic complications, both when therapy is continued and when it is interrupted, though presumably a given thrombotic event might be more severe in an individual who interrupts rather than continues their antithrombotic therapy. This nuance is challenging to define from retrospective data, and the small number of events and limited group size preclude meaningful conclusions regarding the effect of medication interruption on ischemic risk.

Existing evidence strongly supports that patients on antithrombotic therapy are at increased baseline risk for thrombotic or ischemic complications due to their underlying cardiovascular or thromboembolic conditions [[16], [17], [18], [19]]. These findings are further supported by a recent retrospective cohort study using a large national administrative claims database, which identified active anticoagulant prescription (though did not specify whether therapy was held or continued) as a risk factor for overall complications following cervical epidural steroid injections, noting 3.8 to 5.4-fold increased odds of complications. This association was primarily driven by an increased risk of medical complications (including both thromboembolic events and systemic events such as infection, renal failure, or heart failure) rather than procedural complications, leading the authors to conclude that anticoagulant use likely reflects greater underlying medical comorbidity rather than procedural risk. Notably, anticoagulant or antiplatelet prescription was not associated with epidural hematoma in this study [42].

Furthermore, literature specific to interventional spine procedures has found that interrupting anticoagulant agents pre-procedurally has been associated with increased risk of ischemic complications. For example, in a prospective study of 1886 patients undergoing spinal and non-spinal procedures whose warfarin was temporarily interrupted, there was a 0.48% risk (95% CI 0.2—0.9) of catastrophic thromboembolic cerebrovascular or cardiopulmonary events. These nine events included cases of stroke, myocardial infarction, pulmonary embolism, and death [21]. Similar findings have been reported across broader medical and perioperative settings, where interruption of antithrombotic therapy has been associated with increased rates of ischemic stroke and major adverse cardiovascular events [[22], [23], [24], [25],^51^,^52^]. Additionally, emerging evidence suggests a transient period of increased thrombotic risk following discontinuation, potentially related to rebound hypercoagulability [26].

In the larger context, recognition of the low risk of hemorrhagic complications and the relatively elevated risk of ischemic complications has contributed to changing guidelines. This is combined with advances in procedural technique, including the routine use of fluoroscopic guidance, contrast injection, and preprocedural imaging review, which have all substantially improved the safety profile of cervical transforaminal injections [4,53]. The International Pain and Spine Intervention Society (IPSIS) 2024 guidelines have categorized CTFESI procedures as presumed low-intermediate bleeding risk with the caveat that this recommendation is limited by insufficient data [40].

Altogether, these findings and the changing landscape emphasize the importance of considering antithrombotic therapy management on a case-by-case basis, carefully weighing the risks and benefits. For example, certain indications are associated with higher risk of thromboembolic complications, including mechanical heart valves, high CHA_2_DS_2_-VASc scores, recent venous thromboembolic events, severe thrombophilia, or recent coronary stenting [19,[54], [55], [56]]. Alternatively, factors associated with increased bleeding risk in interventional spine procedures include advanced age, underlying coagulopathy, or renal impairment [19,57]. The recently updated 5th edition of the American Society of Regional Anesthesia and Pain Medicine (ASRA Pain Medicine) guidelines on the management of antithrombotic and thrombolytic therapy for regional anesthesia emphasize the importance of individualized risk assessment. The guidelines incorporate a suggested thromboembolic risk stratification scheme (high, moderate, or low) based on patient-specific risk factors, adapted from the 2022 CHEST guidelines [19,57].

Our study had several limitations. As a retrospective chart review, our study is subject to potential inaccuracies in documentation, selection bias, and confounding. Hemorrhagic and ischemic complications are rare events, and our small sample size and low complication rates resulted in limited statistical power to detect differences between groups or compare relative risk of complications. This study was conducted at a single academic health care system, which limits the generalizability of findings. Additionally, it is possible that patients had complications that were not detected due to presenting to other health systems, although it is unlikely that the interventionalist was not made aware of a major complication.

Future studies should aim to compare the relative risk of ischemic versus hemorrhagic complications in CTFESI and CSNRB procedures among those continuing versus interrupting antithrombotic therapy, which requires a larger sample size. Additionally, future larger-scale studies are needed to strengthen the evidence regarding procedural risk.

## 5 Conclusion

This study describes the observed incidence of hemorrhagic and ischemic complications following interruption versus continuation of antithrombotic therapy among patients who underwent CTEFSI and CSNRB procedures. No hemorrhagic complications were observed in either group. Regarding ischemic complications, two events occurred among 80 procedures in the interrupted group and one event among 25 in the continued group. However, the small sample size and low complication rates limit our ability to draw meaningful conclusions regarding the safety of continuing antithrombotic therapy or the comparative risk of interrupting therapy. These findings add descriptive data to the limited literature regarding periprocedural antithrombotic management for cervical transforaminal procedures. Larger studies are needed to better characterize the relative risks and inform individualized management.

## Funding

This study was supported by an investigator-initiated research grant from the Skaggs Research Foundation (paid directly to the University of Utah).

## Declaration of competing interest

The authors declare that they have no known competing financial interests or personal relationships that could have appeared to influence the work reported in this paper.
